# The association between diabetes and thyroid cancer risk: a hospital-based case-control study in China

**DOI:** 10.1186/s12902-021-00684-y

**Published:** 2021-01-28

**Authors:** Meng Wang, Wei-Wei Gong, Feng Lu, Ru-Ying Hu, Qing-Fang He, Min Yu

**Affiliations:** Zhejiang Provincial Center for Disease Control and Prevention, 3399 Binsheng Road, 310051 Hangzhou, China

**Keywords:** Thyroid cancer, Diabetes Mellitus, Case‐control study

## Abstract

**Background:**

Previous studies have indicated inconsistent relationships of diabetes with thyroid cancer risk, yet little is known in China. In this study, we aimed to investigate the associations between diabetes, diabetes duration and the risk of thyroid cancer in Chinese population.

**Methods:**

A 1:1 matched case-control study was performed between 2015 and 2017 in Zhejiang Province including 2,937 thyroid cancer cases and 2,937 healthy controls. Odds ratios (ORs) with 95 % confidence intervals (CIs) for thyroid cancer were estimated in logistic regression models. Specific effects stratified by age, as well as sex, body mass index (BMI) and family history of diabetes were also examined.

**Results:**

Overall, neither diabetes (OR = 0.75, 95 % CI: 0.21–2.73) nor diabetes duration (OR = 0.14, 95 % CI: 0.02–1.22 for diabetes duration ≦ 5 years; OR = 2.10, 95 % CI: 0.32–13.94 for diabetes duration > 5 years) was significantly associated with thyroid cancer. In stratified analyses, significant lower risk of thyroid cancer was observed among subjects with diabetes and shorter diabetes duration ( ≦ 5 years), but limited to those who were aged more than 40 years, female, overweight/obese and had positive family history of diabetes.

**Conclusions:**

Diabetes and shorter diabetes duration were significantly associated with decreased risk of thyroid cancer in individuals characterized by older age, female sex, higher BMI and positive family history of diabetes.

## Background

Diabetes is one of the fastest growing health challenges of the 21st century. Estimated by the International Diabetes Federation, there were currently 463 million adults with diabetes in 2019 and the number was expected to increase to 700 million by 2045 globally [1]. Cancer is also a major public health issue. The World Health Organization reported that there were an estimated 18.1 million new cancer cases and 9.6 million cancer deaths in 2018 worldwide [2]. As the global burden of diabetes and cancer grow, an increasing number of studies have been exploring the interrelationships between them, among which the majority focuses on the occurrence and prognosis of cancer among people with diabetes. Interestingly, elevated incidence and mortality in overall or specific types of cancer in diabetes patients have been reported in some studies [3–6], but not in others [7–9]. Among diabetes patients, the inconsistent incident risk also extends to thyroid cancer, which is the most common endocrine cancer [10]. In recent decades, with a significant increase in incidence of thyroid cancer worldwide [11], public health concerns have been raised on the association between diabetes and thyroid cancer risk. Specifically, some studies lend support to the hypothesis that diabetes increases the risk of thyroid cancer [12–14], while other studies report either null or protective effect [15–18]. Along with the inconclusive findings on diabetes-cancer effects across the literature, the possible mechanisms linking diabetes and cancer are partially understood. It is widely assumed that the association of diabetes with cancer is due to the hyperglycemia, hyperinsulinemia and inflammation [19–21]. Some studies have reported that the shared risk factors (e.g. age, sex, body mass index, etc.) for diabetes and cancer are also involved. Besides, the diabetes duration [22] and treatments [23, 24] may play a contributory role in the link between diabetes and cancer, which still needs further study to verify.

## Methods

### Study subjects

With a 1:1 matched (by age and sex) hospital-based case-control study, we aimed to explore the associated factors of thyroid cancer in Zhejiang Province. The study design and subjects selection have been described in detail elsewhere [25, 26], and are thus only briefly recounted here. Totally, 2,937 case subjects were selected from thyroid cancer patients diagnosed in local hospitals and were identified by physician review of medical records and pathology reports. Besides, based on ICD-10 (malignant neoplasm of thyroid gland [C73]), the cases were further identified. In the same area and same year, 2,937 control subjects were selected from thyroid- healthy examinees who underwent the annual routine physical examination in local hospitals.

### Questionnaire

According to a designed questionnaire, the interviewer collected the socio-demographic characteristics, individual history and family history of chronic diseases, lifestyle behaviors, environmental hazardous substances exposure, dietary habits and other information face-to-face at enrollment. The process of questionnaire design and specific contents of questionnaire have been described in detail elsewhere [25].

### Definition of the exposure variables

In this study, self-reported diabetes and diabetes duration were the exposure of interests. The diabetes status (yes, no) was based on a questionnaire item asking about whether the subjects had ever been diagnosed with diabetes by a doctor. In this study, we did not collect the information on the diabetes drugs taken by the subjects. Diabetes duration was calculated from the year of diabetes diagnosis to the year of thyroid cancer diagnosis in the case subjects and from the year of diabetes diagnosis to the year of thyroid ultrasound screening in the control subjects, respectively. In this study, the diabetes duration was grouped as no diabetes, ≦5 years, and > 5 years.

### Definition of the covariates

Consistent with the existing literature and the definition of confounding factors, the following variables was collected at enrollment and chosen as the potential confounders, which included age, sex, education level, average monthly household income, smoking, alcohol drinking, body mass index (BMI), annual X-ray examination, family history of diabetes, family history of thyroid cancer. Notably, smoking was assessed by the questionnaire item: “How often do you smoke now?” (never, ever smoked but have quitted, currently smoked but not daily, currently and daily). Subjects answered ever smoked but have quitted were considered as ever smokers and those answered currently smoked daily / non-daily were considered as current smokers. Alcohol drinking was assessed by the questionnaire item: “During the past 12 months, how often did you drink any alcohol?” (never, only occasionally, only at certain seasons, every month but less than weekly, usually at least once a week). Subjects answered alcohol drinking only occasionally and at certain seasons were classified as occasional and those drank alcohol every month weekly or less than weekly were classified as current regular. Besides, BMI was calculated based on the Chinese adult BMI classification [27].

### Statistical analysis

The associations between socio-demographic characteristics and thyroid cancer were compared with Chi-square tests (Table 1).

**Table 1:** Socio-demographic characteristics of subjects and their relationships with thyroid cancer

Factors	Cases (*N =* 2937; *%*)		Controls (*N =* 2937; %)	P
Age (Years)	49.27 ± 1.19		49.15 ± 1.20	Matched
> 40	2264 (77.1)		2242 (76.3)	
≤ 40	672 (22.9)		692 (23.6)	
Sex				Matched
Males	676 (23.0)		676 (23.0)	
Females	2261 (77.0)		2261 (77.0)	
Education level				0.86
No formal/primary school	1154 (39.3)		1146 (39.0)	
Middle/high school	1231 (41.9)		1250 (42.6)	
College/university/postgraduate	546 (18.6)		534 (18.2)	
Average monthly household income (Yuan)				0.01
≤ 2000	2127 (72.4)		2060 (70.1)	
2000–5000	343 (11.7)		323 (11.0)	
> 5000	464 (15.8)		548 (18.7)	
Smoking category				0.75
Never	2543 (86.6)		2550 (86.8)	
Ever and current	394 (13.4)		387 (13.2)	
Alcohol category				< 0.001
Never	2179 (74.2)		1907 (64.9)	
Occasional	542 (18.5)		716 (24.4)	
Current regular	216 (7.4)		314 (10.7)	
Body mass index				< 0.001
Normal weight	1594 (54.3)		1793 (61.0)	
Underweight	91 (3.1)		141 (4.8)	
Overweight	929 (31.6)		804 (27.4)	
Obese	223 (7.6)		198 (6.7)	
Annual X-ray examination				< 0.001
0 time	1155 (39.3)		1617 (55.1)	
≥ 1times	1782 (60.7)		1320 (44.9)	
Family history of diabetes				< 0.001
Negative	458 (15.6)		285 (9.7)	
Positive	153 (5.2)		166 (5.7)	
Family history of thyroid cancer				0.001
Negative	528 (18.0)		412 (14.0)	
Positive	83 (2.8)		32 (1.1)	

Percentages of each variable may not equal 100 because of missing data or rounding.

**Fig. 1:**
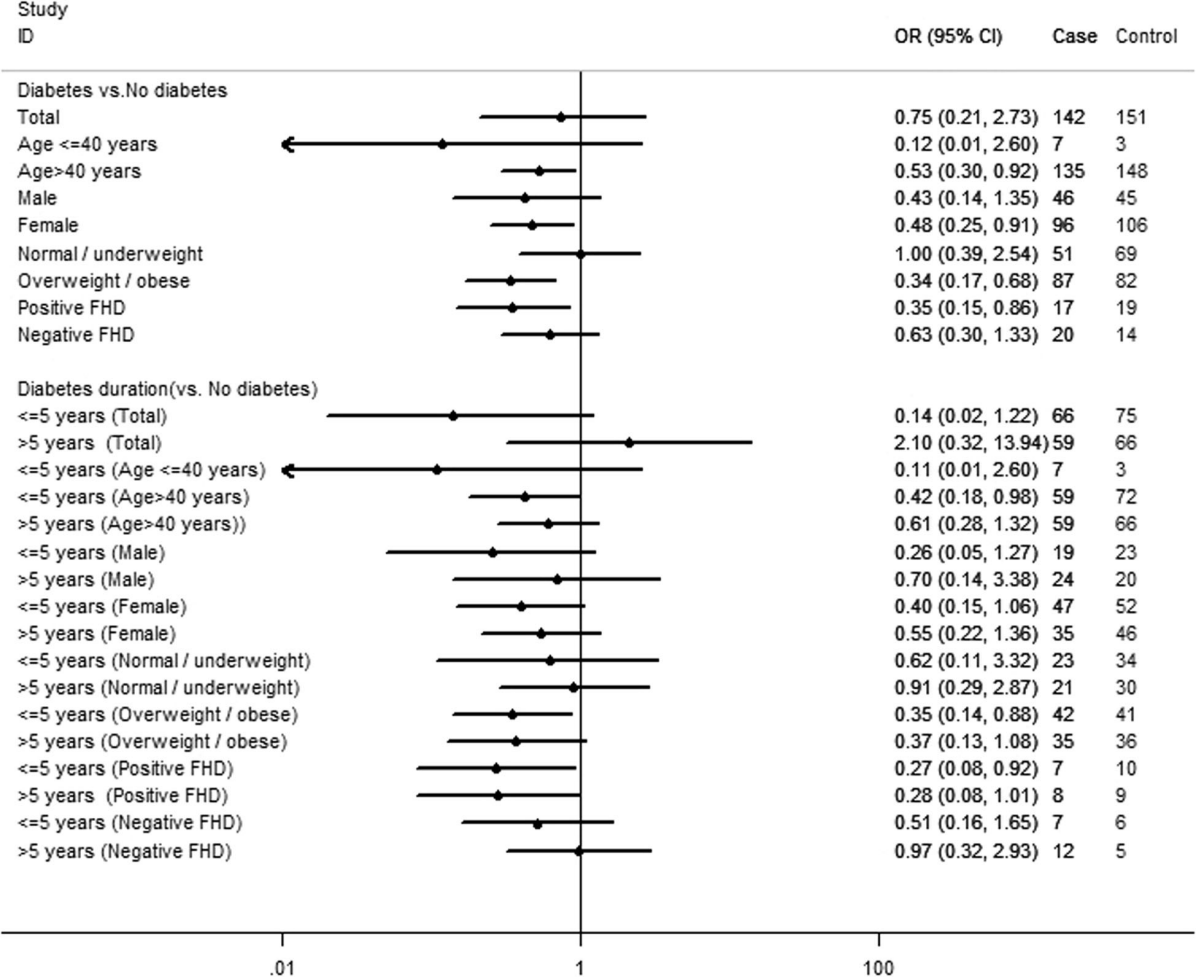
Associations between diabetes, diabetes duration and thyroid cancer in total subjects and stratified by age, as well as sex, body mass index (BMI) and family history of diabetes (FHD). OR odds ratio, CI confidence interval. ORs were adjusted for all variables listed in Table 1

To examine the associations between diabetes, diabetes duration and thyroid cancer risk in total subjects, we used multivariate conditional logistic regression models with adjusting for covariates. Furthermore, to compare the associations between diabetes, diabetes duration and thyroid cancer risk among subjects with different characteristics of age, sex, BMI and family history of diabetes, we also performed relevant stratified analyses. For this part of analyses, multivariate logistic regression models were conducted with adjusting for the covariates. The specific results were showed in Fig. 1.

The associations of diabetes, diabetes duration with thyroid cancer risk were reported by odds ratios (ORs) with 95 % confidence intervals (CIs). All statistical tests were based on the 2-sided 5 % level of significance using SAS statistical package (version 9.2, SAS Institute, Inc., Cary, NC, USA).

## Results

### General information of study subjects

A total of 2,937 pairs of subjects participated in the study. Matched by age and sex, the mean age in case and control subjects was 49.27 ± 1.19 years and 49.15 ± 1.20 years, respectively. Among them, there were 2,261 pairs (77.0 %) of females and 676 pairs (23.0 %) of males. In this study, 293 subjects reported that a doctor had told them they had diabetes. Frequencies and proportions of socio-demographic characteristics among case and control subjects are shown in Table 1. Through comparison, the cases and control had significant differences in the characteristics of average monthly household income (*P* = 0.01), alcohol category (*P* < 0.001), BMI (*P* < 0.001), annual X-ray examination (*P* < 0.001), family history of diabetes (*P* < 0.001) and family history of thyroid cancer (*P* = 0.001).

### The association of diabetes with thyroid cancer risk

Overall, we found no significant association between diabetes and thyroid cancer risk (OR = 0.75, 95 % CI: 0.21–2.73). In stratified analyses, compared to subjects without diabetes, significant lower risk of thyroid cancer was seen in subjects with diabetes, but limited to those who were aged more than 40 years (OR = 0.53, 95 % CI: 0.30–0.92), female (OR = 0.48, 95 % CI: 0.25–0.91), overweight/obese (OR = 0.34, 95 % CI: 0.17–0.68) and had positive family history of diabetes (OR = 0.35, 95 % CI: 0.15–0.86) (Fig. 1).

### The association of diabetes duration with thyroid cancer risk

In this study, we compared the risk of thyroid cancer for subjects with diabetes duration ≦ 5 years and > 5 years, with reference to subjects without diabetes. Overall, we found no significant risk of thyroid cancer in subjects with diabetes duration ≦ 5 years (OR = 0.14, 95 % CI: 0.02–1.22) and > 5 years (OR = 2.10, 95 % CI: 0.32–13.94). In stratified analyses, significant lower risk of thyroid cancer was seen in subjects with diabetes duration ≦ 5 years, but restricted to those who were aged more than 40 years (OR = 0.42, 95 % CI: 0.18–0.98), overweight/obese (OR = 0.35, 95 % CI: 0.14–0.88) and had positive family history of diabetes (OR = 0.27, 95 % CI: 0.08–0.92) (Fig. 1).

## Discussion

Overall, the findings of the present study suggest no associations between self-reported diabetes, diabetes duration and risk of thyroid cancer, after adjustment for potential confounders. Since consistent with some previous studies showing that diabetes, diabetes duration was not associated with risk of thyroid cancer [15–18], our results may aggravate the heated debate on the potential effects of diabetes on thyroid cancer. Besides, to make our study more comparable with existing literature on this topic, we further conduct the stratified analyses according to the baseline characteristics of age, as well as sex, BMI and family history of diabetes. Based on the findings, a significant lower risk of thyroid cancer was observed among subjects with diabetes and shorter diabetes duration ( ≦ 5 years) within some strata.

There is increasing evidence that the thyroid cancer risk among patients with diabetes is various by sex [28–30] and our study is no exception. In the current study, our results indicate that the risk of thyroid cancer is lower only in females with diabetes compared to females without diabetes. This observed decreased risk of thyroid cancer among women with diabetes is consistent with findings from studies conducted in Korea, as well as in Japan and Italy [17, 31, 32], although some estimates were of borderline significance. The older age and higher BMI were proposed as common risk factors for diabetes and thyroid cancer, while their role in the diabetes-cancer linkage has not been well studied. In this study, we find lower risk of thyroid cancer among patients with diabetes who are aged more than 40 years or overweight/obese. Inconsistently, a recent meta-analysis including 5 US-based prospective cohort studies suggested no statistically-significant results within strata of baseline age (< 60 and ≥ 60 years) and BMI (< 25 and ≥ 25 kg/m^2^) [15]. Furthermore, a population-based cohort study in Italy also investigated the thyroid cancer risk among patients with diabetes by strata of age. After stratification, the study only reported a significant positive association between diabetes and thyroid cancer risk for older patients (65–74 years) [33]. Family history of diabetes is an established risk factor for diabetes [34], which reflects the gene-environment interactions within families. A previous study has reported that a positive family history of diabetes increased the effects of diabetes on overall and specific types of cancer risks, including stomach, rectum, corpus uteri and pancreas [31]. However, due to the limited number of cases, the corresponding effect on thyroid cancer risk was not investigated. According to the analyses, our results reveal that the risk of thyroid cancer is lower in patients with diabetes who had a positive family history of diabetes.

In this study, the underlying mechanisms for the decreased risk of thyroid cancer among patients with diabetes in different strata are not clear. Regarding the sex-specific effect of diabetes on thyroid cancer, one possible explanation is the inverse association between blood glucose level and thyroid cancer risk in women, which has been reported by Metabolic syndrome and Cancer project [35]. Regarding the lower risk of thyroid cancer among overweight/obese patients with diabetes, there is little convincing evidence existing for this observation. In addition, regarding the observed decreased risk of thyroid cancer in this study, another potential factor is the diabetes drugs. Metformin is the most commonly used drug and recent studies have indicated that metformin use in diabetes patients may reduce the risk of thyroid cancer [36, 37]. Regretfully, we did not collect the information about intake of diabetes drugs and thus, their specific effects on thyroid cancer cannot be evaluated in this study.

Recent evidence has suggested that patients with diabetes have a higher risk of cancer in the decade before and shortly after diabetes diagnosis [22]. In this study, we observe lower risk of thyroid cancer in the first five years after diabetes diagnosis, which is in line with results from studies conducted in Korea [17] and Taiwan [16]. As for the decreased risk of thyroid cancer in patients with diabetes duration ≦ 5 years, we have no obvious explanation. However, in the study of Taiwan [16], the author proposed that metformin was widely used in patients with early diabetes and the protective effect of metformin against thyroid cancer may account for such a finding.

Our study has several strengths. This study is one of the few studies investigating the specific associations between diabetes, diabetes duration and thyroid cancer among individuals with different characteristics in mainland China. It is a hospital-based 1:1 case-control study with relatively large sample of 2,937 pairs of subjects. All incident thyroid cancer cases are diagnosed in hospitals and further identified by physician review of medical records and pathology reports, which will minimize the probability of misclassification.

However, some limitations are also observed. Firstly, the number of subjects with diabetes is relatively small in the study, which could decrease the statistical power to investigate the associations with thyroid cancer, especially in the stratified analyses. Secondly, due to the fact that the related information of diabetes and other variables is collected via self-reporting but not confirmed in the medical records, recall bias may be inevitable in this study. However, evidence was emerging that there was a substantial agreement in determinations of diabetes status by self-reports and those based on actual diagnoses [38]. Meanwhile, indeed, the method of self-reporting may decrease the reliability of findings with limiting our ability to conduct the study based on clinic classification of diabetes. Thirdly, although some potential confounders have been considered in this study, the fact we did not collect information on the diabetes treatment, to some extent, may also affect reliability of the study.

## Conclusions

Although there is no association between overall diabetes and thyroid cancer, our study suggest that diabetes and short diabetes duration are significantly associated with decreased risk of thyroid cancer among individuals characterized by older age, female sex, higher BMI and positive family history of diabetes. These findings have certain clinical and public health implications for early identification of individuals who are at higher risk for thyroid cancer and providing support for the development of thyroid cancer preventive strategies among patients with diabetes.

## Data Availability

The data will be available upon reasonable request from the corresponding author.

## Abbreviations

OR: Odds ratio
CI: Confidence interval
BMI: Body mass index
CDC: Center for Disease Control and Prevention
